# Components of a healthy diet and different types of physical activity and risk of atherothrombotic ischemic stroke: A prospective cohort study

**DOI:** 10.3389/fcvm.2022.993112

**Published:** 2022-10-14

**Authors:** Anna Johansson, Stefan Acosta, Pascal M. Mutie, Emily Sonestedt, Gunnar Engström, Isabel Drake

**Affiliations:** ^1^Department of Clinical Sciences in Malmö, Lund University, Malmö, Sweden; ^2^Department of Cardiothoracic and Vascular Surgery, Vascular Center, Skåne University Hospital, Malmö, Sweden

**Keywords:** healthy diet, physical activity, lifestyle, atherothrombotic ischemic stroke, cohort study, epidemiology

## Abstract

**Background:**

Diet and physical activity (PA) are modifiable risk factors thought to influence the risk of ischemic stroke (IS). However, few studies have examined their effect on different subtypes of IS.

**Aim:**

To examine components of overall diet quality and different types of PA in relation to the risk of atherothrombotic IS (aIS).

**Materials and methods:**

The study population included 23,797 participants (mean age 58 years; 63% women) from the Malmö Diet and Cancer Study cohort. Participants were enrolled between 1991 and 1996 and followed until end of 2016 (median follow-up 21.5 years). Incident aIS events were identified using national registries (total cases 1,937). Measures of PA (total, leisure-time, occupational, and domestic) were assessed using a baseline questionnaire and dietary intakes were estimated using a modified diet history method. Overall diet quality was assessed using a diet quality index. Intake of key food groups and beverages associated with overall diet quality were investigated separately. Hazard ratios (HR) and 95% confidence intervals (CI) were estimated using multivariable Cox regression models adjusting for confounders.

**Results:**

A high diet quality with high intake of fruit and vegetables, fish and shellfish and low intake of sugar-sweetened beverages and red and processed meat compared to a low diet quality was associated with lower risk of aIS (HR = 0.82, 95% CI = 0.69–0.97; *p* = 0.015). Leisure-time PA was associated with reduced risk of aIS (HR = 0.95 per SD increase in MET-hours/week, 95% CI = 0.91–0.99; *p* = 0.028) with null associations observed for total, occupational and domestic PA level. We observed no significant interaction between diet and PA on the risk of aIS. The standardized 20-year risk of aIS among subjects with low leisure-time PA and low diet quality was 8.1% compared to 6.1% among those with high leisure-time PA and high diet quality.

**Conclusion:**

Several components of a healthy diet and being physically active may reduce the risk of aIS, however, the absolute risk reduction observed was modest. A high diet quality seemed to have a risk reducing effect regardless of level of PA suggesting that individuals with a sedentary lifestyle may still gain some positive health benefits through a healthy diet.

## Introduction

Ischemic stroke (IS) remains a leading cause of death globally (1), and in 2019 7.6 million cases of IS occurred (2). Additional consequences of IS include suffering and disabilities among its survivors as well as great costs for health care systems worldwide. Apart from smoking, potential modifiable risk factors for IS include diet and physical activity (PA) (3). The role of specific dietary factors and type and level of PA in relation to risk of IS are, however, not fully established and further knowledge is needed to assess the potential utility of modification of such risk factors on the development of the disease. IS can arise from clots formed in atherosclerotic large or small vessels, atherotrombotic stroke (aIS), or the source can be cardioembolic due to atrial fibrillation or flutter (AF) (4). Large vessel disease and small vessel disease have been suggested to share risk factors such as diabetes mellitus and smoking, whereas cardioembolic stroke differs in its risk profile (5) as well as severity (6), which further strengthens the rationale to separately evaluate risk factors for stroke subtypes. We hypothesize that the role of diet and PA in the development of the different IS subtypes could potentially differ and examining aIS separately from cardioembolic IS may thus clarify putative associations.

According to the World Health Organization PA plays a major role in prevention of non-communicable diseases, and is important for general good health (7). They recommend ≥150 mins of moderate-intensity aerobic PA per week in order to gain health benefits (7). An inverse relationship between PA and risk of IS has previously been shown, but the effect of different modes as well as the amount of PA needed to maximize risk reduction for IS is less clear (8). Further, different types of PA have been shown to have different effects on the risk of IS. Higher levels of occupational PA have been associated with increased risk of stroke, while leisure-time PA associates with reduced risk of stroke (7). Dietary patterns as well as specific foods and beverages have been associated with risk of cardiovascular disease (CVD) (9). A recent systematic review found strong evidence that dietary patterns such as a Mediterranean diet and The Dietary Approaches to Stop Hypertension diet as well as a high intake of nuts, olive oil, fish and a moderate intake of low-fat milk and dairy products contribute to reducing the risk of stroke (10). In line with these findings, a previous study within the Malmö Diet and Cancer Study (MDCS) found that a data driven health-conscious food pattern defined by high intake of fruit and vegetables, fermented milk and low intake of sugar-sweetened beverages and red and processed meats was associated with a decreased risk of IS (11). In a recent study, a high diet quality and higher level of leisure-time PA predicted lower risk of aIS independent of established risk factors (12). The present study aimed to investigate the putatively causal nature of the associations between diet quality and PA by structured assessment of included model covariates. In addition, specific aspects of diet quality as well as different types of PA (total, leisure-time, occupational, and domestic) were examined in relation to aIS to further understand the underlying nature of the previously observed associations within this cohort. A healthy lifestyle typically includes aspects of both diet and PA. We hypothesized that these modifiable risk factors independently affect risk of aIS. To test this hypothesis, we examined the joint effect of diet quality and PA on risk of aIS.

## Materials and methods

### Study design, setting, and population

The MDCS cohort includes 30,446 men and women born in Malmö between 1923 and 1945. Participants were recruited to baseline examinations between 1991 and 1996 which included an extensive self-administered questionnaire (13) and a dietary assessment using a modified diet history method (14). Blood pressure and anthropometric measures were directly measured, and blood samples were drawn by project nurses. Subjects with prevalent IS (any subtype) (*n* = 233), AF (*n* = 312), diabetes mellitus (*n* = 1,380), atherosclerotic disease (coronary artery disease, peripheral artery disease, and carotid artery disease) (*n* = 785), and subjects with missing data on included covariates (*n* = 4,739) were excluded. The final study population included 23,797 participants (63.2% women) (Supplementary Figure 1). The study was conducted ethically in accordance with the World Medical Association Declaration of Helsinki. The protocol was approved by the Regional Ethical Review Board in Lund, Sweden (Dnr §LU 51-90, 2007/166). Written and oral informed consent for inclusion and publication was given by all subjects prior to participation (15).

### Endpoint ascertainment

Participants with a diagnosis of IS according to the Swedish revision of the International Classification of Disease (ICD) version 8 (433, 434), version 9 (434, 436), and version 10 (I63, I64) were identified using the Swedish National Patient register and the Cause of Death Register Participants. The validation of IS diagnosis has been described in detail previously (12). In short, based on a random sample of 100 patients, the diagnosis of IS was confirmed in 89% of the sample. All subjects were followed from baseline examination until date of first IS event, emigration from the country (<0.5%), death or 31 December 2016, whichever occurred first. Further, subjects diagnosed with AF within 30 days of their IS were censored at their date of diagnosis of incident AF (ICD8-427.9, ICD9-427D, and ICD10-I48) and excluded from the endpoint.

### Assessment of physical activity level

Information on PA level was gathered through the self-reported baseline questionnaire. To estimate individual PA level, intensity factors [the ratio of work metabolic rate to resting metabolic rate; metabolic equivalent task (MET)] were assigned to each specific activity reported as previously described (16, 17). Three main domains of PA were estimated as MET-hours/week: leisure-time PA, occupational PA, and domestic PA. Leisure-time PA was assessed based on reported number of minutes/week for seventeen different leisure-time PA, each assigned a different MET factor (17). Occupational PA was assessed for participants currently working and based on hours of work per week multiplied by a specific MET-factor representing self-reported physical intensity at work (17). Domestic PA was assessed based on reported hours of household work per week which was assigned a MET-factor of 2.5 regardless of type of household work performed (16). Total MET-hours/week was generated by adding the number of reported MET-hours per week from leisure-time PA, occupational work, domestic work, time for self-care, sleep, and passive time. Time for self-care was defined as a constant of 1 h (1.6 MET) per participant. Sleeping time was only reported in a subset of participants (1,706 women and 1,188 men). For each participant the median sleep time (7.3 h/day) was used and given a MET factor of 0.9 (13, 16). Passive time (i.e., time not spent being active or asleep) was given a MET factor of 1.3. The total individual PA level was defined as the hourly PA rate (total MET-hours per week/168) (13, 14).

### Assessment of diet quality

Overall diet quality was assessed using a diet quality index based on adherence to the Swedish nutrition recommendations and food-based dietary guidelines from 2005 (18) that was previously developed specifically for the MDCS cohort (19). The index includes six components: Saturated fatty acids (SFA) ≤ 14 energy (E)%, polyunsaturated fatty acids 5–10 E%, fish and shellfish ≥ 300 g/week, sucrose ≤ 10 E%, dietary fiber ≥ 2.4 g/megajoule (MJ), and fruit and vegetables ≥ 400 g/day. Reaching the pre-defined cut-offs results in one point per component, with a maximum score of six points. For descriptive analysis, participants were categorized as having a low (0–1 points), medium (2–4 points), or high (5–6 points) diet quality. In order to assess specific aspects of diet quality that may be important in development of aIS, we considered food groups and beverages that have been associated with atherosclerotic disease as well as stroke (10, 20, 21) and have been addressed in many national dietary guidelines (22). The association between intake of each of the dietary items with overall diet quality was analyzed. Specific nutrients were not included since such intake levels may reflect a range of food groups with potentially differing effect and are thus less optimal in dietary guidance for the general population. Thus, the examined dietary items included fruit and vegetables, high-fiber bread and cereals, fish and shellfish, red and processed meats, sugar-sweetened beverages (SSB), coffee, low-fat milk products, fermented milk products, and high-fat dairy products. Total intake of high-fat dairy products was assessed as the sum of portions per day per 1,000 kcal of total energy intake and included butter, regular-fat alternatives (≥2.5% fat) of milk, yogurt, and sour milk; cream (>12% fat); and regular-fat cheese (>20% fat). Portions were used to analyse the sum of dairy products due to varying water content and because such foods are usually consumed in different weights (e.g., cheese vs. milk). Standard portion sizes from the MDC study or National Food Agency in Sweden were used as follows: milk and yogurt (200 g/portion), cheese (20 g/portion), cream (25 g/portion), ice cream (75 g/portion), and butter (7 g/portion) (11). All other dietary intakes (g/day) were expressed as relative intakes (g/day/1,000 kcal) in order to adjust for total energy intake.

### Assessment of covariates

**FIGURE 1:**
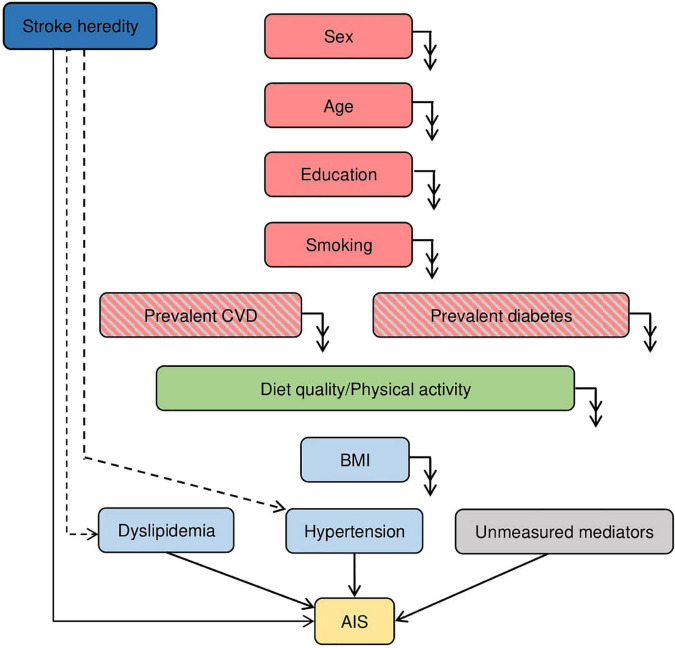
Directed Acyclic Graph (DAG) derived from the literature and expert knowledge of the MDCS cohort. Nodes represent variables and arrows represent causal associations. Double arrows indicate effect on all downstream variables. Dashed arrows indicate potential effect. Red colored variables represent confounding factors, light blue colored variables represent mediating factors, light gray colored variables represent mediating factors not included in study and dark blue colored variables represent competing exposure for the association between diet quality and PA (exposure, green colored) on the risk of atherothrombotic ischemic stroke (AIS, outcome, yellow color). Red and gray colored variables are excluded in the present study.

Covariates considered for inclusion in multivariable models were identified using a directed acyclic graph (DAG) (Figure 1). The online software DAGitty (23) was used to identify the minimal adjustment sets in order to assess the total and direct effects of dietary and PA exposures on risk of aIS. DAG evaluation suggested the following confounders (i.e., common causes) for the association between diet and PA on aIS: age, sex, educational level, and smoking. Identified mediating variables available in the current study included BMI, hypertension, and dyslipidemia. There were, however, several potential mediating factors that were unavailable for the current study (i.e., unmeasured mediating factors) such as blood glucose (24) and low-grade inflammation (25). Further, stroke heredity was identified as a potential competing exposure and was adjusted for to improve precision. Since diet and PA are interrelated variables with unclear direction of effect, we mutually adjusted for both in the model when aiming to estimate the direct effect of these variables on aIS.

#### Confounders

Information on age and sex was extracted from the participants’ Swedish personal identification number. Educational level was defined as less than 9 years, elementary school (9–10 years), upper secondary school (11–13 years), university without a degree, and university degree. Smoking was categorized as never, former, or current.

### Mediators

Weight (kg) and height (cm) were measured at baseline examinations and used to calculate body mass index (BMI; kg/m^2^). Hypertension was defined as systolic blood pressure ≥ 140 mmHg, diastolic blood pressure ≥ 90 mmHg or current use of antihypertensive medications. Dyslipidemia was defined as having an ApoB/ApoA-I ratio of >0.9 for men and >0.8 for women or reporting current use of lipid-lowering drugs in the baseline questionnaire (12).

### Competing exposure

Stroke heredity was identified as a potential competing exposure but may also have an effect on important mediating risk factors for aIS (i.e., hypertension and dyslipidemia). We therefore adjusted for stroke heredity when estimating the direct effect of diet and PA on aIS since potential bias may be introduced when adjusting for these mediators. The stroke heredity score was constructed based on participants’ self-reported family history of stroke (mother, father, or sibling with stroke). Participants with no first-degree relatives with stroke were categorized as having a low heredity for stroke while participants with at least two first-degree relatives were categorized as having high heredity for stroke (12).

### Statistical analysis

Baseline characteristics across overall diet quality and total PA level were examined as mean (standard deviation, SD) or median (interquartile range, IQR) for continuous variables and as percentage of total count for categorical variables. Differences across categories of diet quality and total PA level were tested using ANOVA for normally distributed continuous variables, Kruskal-Wallis for skewed continuous variables and Chi-square test for categorical variables. Kaplan-Meier analysis was used to estimate the probability of remaining free of aIS event during the follow-up period for the low, medium, and high diet quality index and tertiles of leisure-time PA. The association between different measures of diet quality and PA in relation to incidence of aIS was modeled with a Cox proportional hazards model with years of follow-up as the underlying time-scale. Hazard ratios (HR) and 95% confidence intervals (CI) were assessed across categories of diet and PA as well as per standard deviation (SD) increase. A basic age- and sex-adjusted model was used in order to evaluate the extent of potential confounding by other variables (i.e., smoking and educational level). The main multivariable model was constructed to assess the total effect of diet and PA measures on risk of aIS and included adjustment for age, sex, educational level, and smoking. An additional multivariable model was examined with further adjustment for potential mediating factors (BMI, hypertension, and dyslipidemia), competing exposure (stroke heredity) and with mutual adjustment for diet and PA. The Schoenfeld test was used to assess deviations from the proportional hazard’s assumption. No significant deviations were detected for the presented models (all *p* > 0.05). Potential effect modification by the included covariates on the associations between diet and PA measures with aIS were assessed by including the multiplicative interaction terms in the examined models. No significant effect modification by any of the included variables was detected (all *p* values > 0.10). To visualize the dose-response association between examined dietary variables and PA we fitted restricted cubic splines using the median level of PA as the reference point and with four knots placed at Harrell’s recommended percentiles. For variables with a substantial proportion of zero reporters (SSB, fermented milk products, and occupational PAL), three knots were used, and the reference point was set to zero. The likelihood ratio test was used to test for significant deviations from linearity (all *p* > 0.05). Sensitivity analysis was performed by excluding potential energy mis-reporters (*n* = 4,331). Energy mis-reporters were identified based on the agreement of the reported total energy intake to basal metabolic rate ratio and the individual PA level estimated for each participant based on time spent on each of the activities described above as well as accounting for time spent sleeping, time for self-care and remaining “passive time” (16). Finally, after exclusion of energy mis-reporters, the joint effect of diet and PA on risk of aIS was examined and tested for interaction by including the multiplicative interaction term in the fully adjusted Cox regression model (adjusting for age, sex, educational level, smoking status, stroke heredity, BMI, hypertension, and dyslipidemia). The corresponding 20-year absolute risk estimates standardized to the mean of all included covariates were estimated across categories of diet and PA.

All statistical analyses were performed using IBM SPSS Statistics (Version 26, Chicago, IL, USA), Stata/SE (Version 15.0, College Station, TX, USA), and R (Version 4.0.2, The R Foundation for Statistical Computing). All tests were two-sided and *p* < 0.05 was set as the level of statistical significance.

## Results

### Description of study population

After a median of 21.5 years of follow-up (IQR = 17.6–23.2), 1,937 (8.1%) participants were diagnosed with aIS. Baseline characteristics across diet quality categories are presented in Table 1 and across total PA level tertiles in Table 2. Most baseline characteristics differed by diet quality and total PA level. A high diet quality was associated with a higher intake of fruit and vegetables, fish and shellfish, high-fiber bread and cereals, low-fat milk products and fermented dairy products, but lower intake of SSB, coffee, red and processed meats, and high-fat dairy products. Descriptive figures of the examined dietary and PA exposures including histograms, bivariate scatter plots and the bivariate correlation coefficients are shown in Supplementary Figure 2.

**TABLE 1:** Baseline characteristics across categories of diet quality.

		Diet quality		
	Low	Medium	High	*p* value*
Number of subjects	3,702	17,004	3,091	
Age (years)	57.6 (8.0)	57.7 (7.6)	58.0 (7.3)	0.036
Male sex (%)	34.7	38.4	30.5	<0.0001
University degree (%)	12.4	15.4	16.9	<0.0001
Current smoking (%)	37.5	28.0	16.7	<0.0001
Hypertension (%)	59.2	59.3	61.2	0.12
BMI (kg/m^2^)	25.1 (3.9)	25.6 (3.9)	25.8 (3.9)	<0.0001
Dyslipidemia (%)	26.0	23.2	24.1	0.001
High stroke heredity (%)	3.8	3.6	3.4	0.82
Total PA level, MET-hours/week	270.1 (53.8)	272.4 (52.6)	276.3 (50.9)	<0.0001
Fruit and vegetables (g/day/1,000 kcal)	123.0 (54.8)	173.8 (89.5)	279.4 (100.5)	<0.0001
Fish and shellfish (g/day/1,000 kcal)	10.5 (8.9)	19.3 (14.7)	30.2 (17.5)	<0.0001
High-fiber bread and cereals (g/day/1,000 kcal**)	12.6 (5.1–22.3)	18.2 (8.8–30.6)	28.7 (17.1–45.3)	<0.0001
Red and processed meats (g/day/1,000 kcal)	52.8 (21.5)	53.0 (22.9)	46.8 (21.8)	<0.0001
Sugar-sweetened beverages (g/day/1000 kcal**)	24.4 (0–80.4)	2.6 (0–39.3)	0.0 (0–22.2)	<0.0001
Coffee (g/day/1,000 kcal**)	208 (119–326)	206 (119–324)	197 (109–316)	0.0052
Low-fat milk products (g/day/1000 kcal**)	55.4 (0–157.1)	76.0 (2.5–160.3)	113.2 (34.5–194.3)	<0.0001
Fermented milk products (g/day/1000 kcal**)	20.7 (0–63.6)	23.6 (0–64.2)	35.3 (0–79.9)	<0.0001
High-fat dairy products (portions/day/1,000 kcal)	1.9 (1.0)	1.7 (0.9)	1.4 (0.8)	<0.0001

Continuous variables are presented as mean (standard deviation, SD) unless otherwise noted. Categorical variables are presented as percentage (%) of total count. *p-values from ANOVA or Kruskal-Wallis for continuous variables and from chi-square test for categorical variables. **Presented as median and interquartile range (IQR).

**TABLE 2:** Baseline characteristics across tertiles of total physical activity (PA) level.

		Total PA level		
	Tertile 1	Tertile 2	Tertile 3	*p* value*
Number of subjects	7,913	7,939	7,945	
Age (years)	59.4 (7.8)	59.2 (8.0)	54.7 (6.1)	<0.0001
Male sex (%)	49.2	26.9	34.3	<0.0001
University degree (%)	13.9	14.5	17.0	<0.0001
Current smoking (%)	28.8	24.8	30.5	<0.0001
Hypertension (%)	63.7	61.7	53.3	<0.0001
BMI (kg/m^2^)	25.8 (3.9)	25.4 (3.9)	25.3 (3.8)	<0.0001
Dyslipidemia (%)	27.8	23.3	20.3	<0.0001
Stroke heredity (%)	3.7	4.1	3.0	<0.0001
High diet quality (%)	10.4	14.7	13.9	<0.0001
Total PA level, MET-hours/week	227.7 (8.0)	253.9 (10.3)	335.8 (42.2)	<0.0001
Leisure-time PA, MET-hours/week	19.1 (10.3)	38.4 (17.9)	38.1 (30.3)	<0.0001
Occupational PA, MET-hours/week**	0 (0–60)	0 (0–60)	140 (105–160)	<0.0001
Domestic PA, MET-hours/week	23.0 (15.1)	45.7 (24.6)	38.4 (29.2)	<0.0001

Continuous variables are presented as mean (standard deviation, SD) unless otherwise noted. Categorical variables are presented as percentage (%) of total count. *p Values from ANOVA or Kruskal-Wallis for continuous variables and from chi-square test for categorical variables. **Presented as median and interquartile range (IQR).

### Overall diet quality and risk of atherothrombotic ischemic stroke

Survival curves by categories of diet quality are shown in Supplementary Figure 3. The association between overall diet quality and risk of aIS is shown in Supplementary Table 1. A high compared to low diet quality was associated with a decreased risk for incident aIS in the total effects model (HR = 0.82, 95% CI = 0.69–0.97; *p* trend = 0.015) and strengthened after exclusion of potential energy mis-reporters (HR = 0.76, 95% CI = 0.63–0.91; *p* trend = 0.0019). After additional adjustments the association remained significant and virtually unchanged (Supplementary Table 1).

### Dietary intakes and risk of atherothrombotic ischemic stroke

**FIGURE 2:**
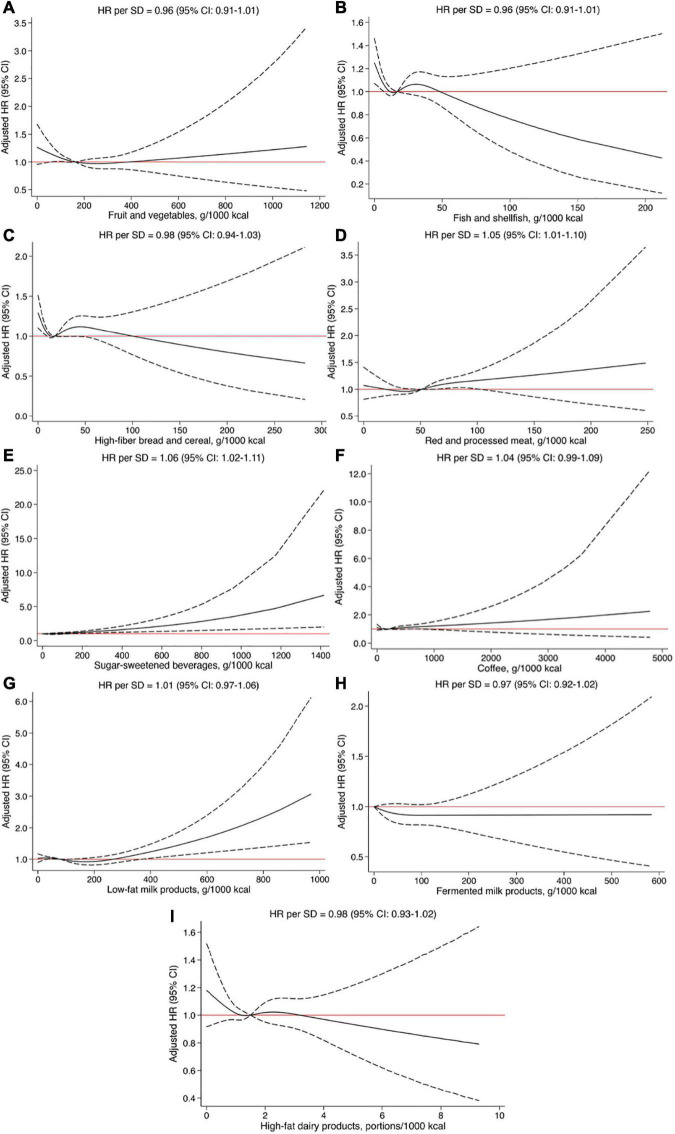
**(A–I)** Restricted cubic splines fitted to Cox regression models showing the associations between dietary intakes and risk of atherothrombotic ischemic stroke. Solid line represents the adjusted hazard ratio (HR) and dashed lines the 95% confidence intervals (CI). All models were adjusted for age, sex, educational level, and smoking status.

The dose-response associations between dietary components with risk of aIS are shown Figures 2A–I. Sensitivity analysis and additional multivariable models as well as HR by quartile of intake are shown in Supplementary Table 1. Total fruit and vegetable intake was not associated with incident aIS (Figure 2A; HR per SD increase: 0.96, 95% CI = 0.91–1.01; *p* value = 0.15). However, after exclusion of potential energy mis-reporters there was a significant protective effect with higher intakes (Supplementary Table 1; HR per SD increase: 0.93, 95% CI = 0.87–0.99; *p* value = 0.019). This association remained significant after adjustment for mediating factors (*p* value = 0.014; Supplementary Table 1). Fish and shellfish intake was not associated with risk of aIS (HR per SD increase = 0.96, 95% CI = 0.91–1.01; *p* value = 0.08) in the main model (Figure 2B). However, the association was significant in sensitivity analysis excluding energy mis-reporters as well as after additional adjustment for mediating factors (Supplementary Table 1). Based on the spline analysis and examining the lack of trend across quartile intake there appeared to be a potential threshold effect, suggesting a higher risk for aIS among subjects with low intake of fish and shellfish, but no added benefit with higher intakes (Figure 2B). High-fiber bread and cereal intake did not associate with risk of aIS (Figure 2C; HR per SD increase: 0.98, 95% CI = 0.94–1.03; *p* value = 0.41). Neither adjustment for mediating factors nor exclusion of energy mis-reporters affected the observed association (Supplementary Table 1). We observed a positive linear association between intake of red and processed meat and risk of aIS (Figure 2D; HR per SD increase: 1.05, 95% CI = 1.01–1.10; *p* value = 0.026). The association was strengthened after excluding energy mis-reporters in sensitivity analysis (*p* value = 0.0026). After adjustment for mediating factors and excluding energy mis-reporters, the association remained significant (HR per SD: 1.07, 95% CI = 1.01–1.13; Supplementary Table 1). Total SSB intake was associated with an increased risk of incident aIS (Figure 2E; HR per SD increase: 1.06, 95% CI = 1.02–1.11; *p* value = 2.5 × 10^–3^). The association remained significant after excluding energy mis-reporters in sensitivity analysis (*p* value = 9.1 × 10^–3^) as well as after additional covariate adjustment (*p* value = 4.7 × 10^–3^; Supplementary Table 1). The association between SSB and aIS was linear, with no indications of any threshold levels (Figure 2E). No associations between coffee intake, low-fat milk products, fermented milk products or high-fat dairy products with risk of aIS (Figures 2F–I and Supplementary Table 1) were observed. These associations were unchanged in sensitivity analysis and after adjustment for mediating factors.

### Physical activity level and risk of atherothrombotic ischemic stroke

**FIGURE 3:**
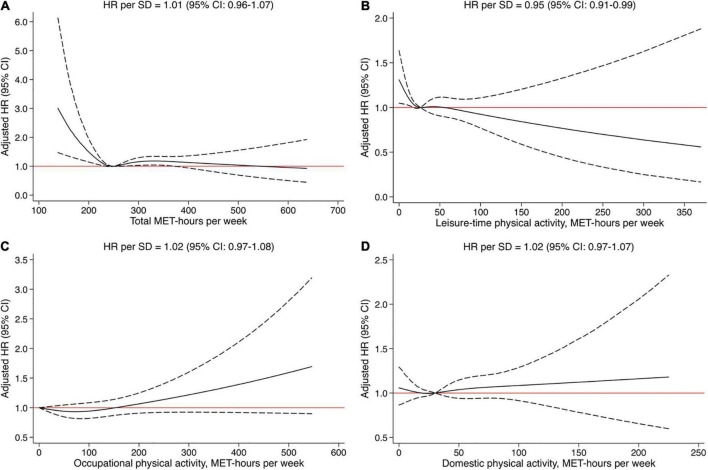
**(A–D)** Restricted cubic splines fitted to Cox regression models showing the associations between physical activity measures and risk of atherothrombotic ischemic stroke. Solid line represents the adjusted hazard ratio (HR) and dashed lines the 95% confidence intervals. All models were adjusted for age, sex, educational level, and smoking status.

Total PA level was not associated with risk of incident aIS (Figure 3A). A decrease in risk was, however, observed to about a total PA level of approximately 240 MET-h/week, beyond which there was no added observed advantage and thus no significant overall linear trend (HR per SD increase: 1.01, 95% CI = 0.96–1.07; *p* value = 0.60; Supplementary Table 2 and Figure 3A). Among the investigated specific measures of PA, there were null associations between domestic and occupational PA with risk of aIS (Supplementary Table 2 and Figures 3C,D). Survival curves by tertiles of leisure-time PA are shown in Supplementary Figure 4. Increasing leisure-time PA (HR per SD: 0.95, 95% CI = 0.91–0.99; *p* value = 0.028; Figure 3B) was associated with a decreased risk of aIS. However, this association was attenuated and not significant in sensitivity analysis excluding energy mis-reporters as well as the model including additional non-confounding covariates (Supplementary Table 2). Similar to total PA level, those with low leisure-time PA (below around 30 MET-hours/week) appeared to be at highest risk, but no significant benefit or harm was seen with higher levels of leisure-time PA (Figure 3B).

### Joint effect of diet quality and leisure-time physical activity level on risk of atherothrombotic ischemic stroke

In Table 3 the joint analyses of diet quality and leisure-time PA level in relation to aIS are shown. No significant effect modification was observed (*p* interaction = 0.82). However, among subjects with low diet quality no effect of increased leisure-time PA was observed, while the trend for protective effect of higher diet quality was observed among all categories of leisure-time PA.

**TABLE 3:** Joint analyses of overall diet quality and leisure-time PA in relation to aIS in the MDCS (*n* = 19,491).

		Diet quality		
Leisure-time PA	Low	Medium	High	*p* trend
Low	1.00 (ref)	0.90 (0.73–1.10)	0.76 (0.54–1.06)	0.15
Subjects/cases	1,150/117	4,249/393	572/47	
Medium	0.99 (0.75–1.31)	0.83 (0.68–1.02)	0.71 (0.52–0.97)	0.026
Subjects/cases	880/88	4,400/378	801/60	
High	1.01 (0.77–1.33)	0.77 (0.63–0.95)	0.77 (0.58–1.03)	0.065
Subjects/cases	774/90	4,165/359	892/76	
*p* trend	0.91	0.044	0.85	*p* interaction = 0.82

HR (95% CI) from a Cox regression model adjusting for age, sex, educational level, smoking status, stroke heredity, BMI, hypertension, and dyslipidemia. Potential energy mis-reporters were excluded from the analysis.

The standardized 20-year incidence rates of aIS across diet quality and leisure-time PA level categories corresponding to the HRs displayed in Table 3 are shown in Supplementary Table 3. The 20-year absolute risk among subjects with jointly low diet quality and low leisure-time PA was 8.1% (95% CI = 6.6–9.5) compared to 6.1% (95% CI = 4.7–7.4) among those with both high diet quality and high leisure-time PA.

## Discussion

The main findings of this study are that a diet with low intakes of fruit and vegetables, fish and shellfish and high intakes of red and processed meat and SSBs was associated with increased risk of aIS. Some previously proposed protective factors such as high-fiber bread and cereal and different types of dairy products were not associated with aIS in this study population. Further, there was no convincing evidence for an association between overall PA and aIS, but leisure-time PA was suggestively protective of aIS.

Few studies have investigated the role of diet and PA in subtypes of IS. In line with the present study results, a prospective cohort study found that adherence to a healthy Nordic diet was associated with reduced risk of IS, in particular large-artery atherosclerosis [according to the TOAST classification (26)] but did not study PA in detail (27). The reduced risk of stroke by intake of a plant-based diet has been suggested to be explained by effects on reducing blood pressure, BMI, dyslipidemia, inflammation and improving microvascular function as well as favorable effects of potassium, antioxidants, fiber, and folate (28). Several studies have linked fruit and vegetable consumption to reduced stroke incidence and mortality (29, 30). In line with this, a higher fruit and vegetable intake was associated with lower risk of aIS, a finding that was more convincing after excluding potential energy mis-reporters, in the present study. However, no association between high fiber bread and cereal intake and aIS could be demonstrated, which is in line with several previous studies (20, 21, 31). Nevertheless, a recent prospective study on two cohorts showed an inverse association between whole grain cold breakfast cereal and risk of IS, but not for total whole grain, suggesting different effects of individual whole grain foods (32).

A higher intake of fish and shellfish lowered risk of aIS in this study. The dose-response analysis suggested a potential threshold effect in that very low consumers had an increased risk of aIS compared to more habitual consumers. Similar associations between fish intake and IS have been found in other populations (33–35). Fish has been suggested to have antithrombotic and anti-inflammatory properties, possibly due to its polyunsaturated fatty acids contents (34).

Intake of red and processed meat was associated with increased risk of incident aIS. Similar results have been found for IS (not hemorrhagic stroke) in a recent meta-analysis of five prospective cohort studies (36–38). In contrast to previous beliefs, studies have shown relatively neutral cardiovascular effects of one of the components of meat; SFA, and instead adverse effects of other components (21). There is now increasing recognition that red and processed meats may have a detrimental effect on cardiometabolic health partly due to its high sodium content causing high blood pressure, a major risk factor for stroke (10). Another component is nitrite, which is believed to promote atherosclerosis and vascular dysfunction. Processed and unprocessed red meat are also sources of heme iron, which might promote oxidative stress and thereby increase the risk of stroke (39).

In our study, SSB intake was associated with increased risk of aIS, consistent with a study on the same population with total stroke as endpoint (40). Studies on risk of subtypes of stroke are limited but several studies have shown associations between SSB intake and increased hypertension, obesity, and dyslipidemia (41, 42). SSB contains high levels of fructose, which has been suggested to cause endothelial dysfunction and sodium retention, leading to high blood pressure. In addition, fructose affects inflammatory factors that plays a role in the development of atherosclerosis (41).

For coffee we found no association with risk of aIS. In contrast to this finding, previous studies have found that a moderate to high intake of coffee reduces the risk of total stroke and IS (43, 44). The lack of association between coffee intake and aIS may be due to reduced variability in coffee intake in the current study population.

For high-fat and fermented milk products we found no association with risk of aIS. Previous studies in the MDCS have found associations between high intake fermented dairy products and decreased risk of CVD as well as stroke (45). Recent studies have found no increased risk of whole-fat milk for obesity, diabetes, or cardiovascular disease in adults and that consuming low-fat dairy products leads to compensation elsewhere in the diet, for example by consuming more carbohydrates (21). This may explain the non-significant tendency for an increased risk of aIS seen among the few individuals with very high intakes of low-fat milk products in our study.

The findings from this study are in line with previous studies linking a DASH-type diet and a Mediterranean-style diet with a decreased risk of IS (46, 47). There is considerable overlap between different definitions of a healthy diet, and the positive benefits of these diets are most likely due to their similarities in focusing on high intakes of healthy food items such as fruit and vegetables and fish and shellfish while reducing intake of unhealthy food items such as SSB and red and processed meat. Additional items related to the Mediterranean diet specifically, such as nuts and olive oil, were not examined in this study because they were not widely consumed within this population of Swedish middle-aged individuals with baseline examinations in the beginning of the 1990s.

A decrease in risk for aIS was observed to about a total PA level of approximately 240 MET-hours/week, beyond this threshold no added advantage was observed, and no significant linear trend was seen for total PA level. Similarly, individuals with low leisure-time PA level appeared to be at higher risk, but no significant benefit or harm was seen with higher levels of leisure-time PA. Another study on the same cohort with CVD as endpoint also concluded that those who were physically inactive have the highest reduction in risk (48). A systematic review and dose-response meta-analysis including 26 studies examining different domains of PA and risk of IS found a significant association between higher total PA level and lower risk of IS. Health benefits declined at total PA level over 50–67 MET-hours/week (49). In this study, a decreased risk for aIS with increasing leisure-time PA was seen when adjusting for confounders, but the association was attenuated after excluding mis-reporters and adjusting for potential mediators. Similarly, a prospective cohort study on 13,069 subjects found an inverse relationship between leisure-time PA and IS as well as non-lacunar stroke (50). The association was attenuated after adjusting for mediators. In contrast to the present study intensity was also taken into consideration. In the present study, participants only reported on type and duration of PA, not level of intensity. Even though using METs assumes the same average intensity for every individual performing a specific activity, it has been shown to be a robust metric, especially for self-reported PA data. Occupational PA was strongly correlated with total MET-hours/week (Supplementary Figure 2), and negatively correlated with both leisure-time PA and domestic PA, suggesting a physically active occupation might leave little room for other types of PA, perhaps with bigger health benefits. Due to the high correlation with total PA level, the impact of occupational PA may have contributed to the non-significant association. While we adjusted for educational level as a marker of socioeconomic status, it is possible that the tendency for an increased risk seen with occupational PA is residually confounded by factors related to socioeconomic status not fully covered by educational level.

No significant interaction was found between diet and PA in the present study. When calculating 20-year standardized risk estimates for aIS we found a lower risk among subjects with a high diet quality, regardless of level of leisure-time PA, suggesting that a healthy diet can provide some health benefits in an inactive lifestyle, but high level of leisure-time PA does not compensate for a low diet quality.

The main strengths of this study include the long follow-up time, the prospective cohort design which reduces the risk of reverse causation, the use of registers to verify incident aIS cases which minimizes bias due to loss of follow-up, and the access to detailed data on diet and PA as well as extensive information on key confounders and mediators. The main weakness of the study is the observational design by which residual confounding cannot be ruled out. The potential causal relationship between diet and PA and aIS was visualized in a DAG (Figure 1). BMI, dyslipidemia, and hypertension were identified as primary mediators. However, several additional factors could potentially be mediating the effect of diet and PA on risk of aIS including low-grade inflammation and blood glucose homeostasis. Since these factors were not available for the current study population, it was not possible to adequately assess the direct effect of diet and PA on aIS. For this reason, the total effect model was presented as the main model when reporting the findings. In addition, the DAG assumes a perfect setting with no measurement error. Several included covariates were self-reported and thus residual confounding may still play a considerable role. The current study population was followed-up for a substantial period of time, but exposures were assessed at baseline only, which allows for a change in diet and PA habits to go unnoticed. Such a change in classification over time would probably lead to non-differential misclassification, which would tend to bias the observed associations toward the null. The risk of residual confounding could have been reduced if the measurements were reiterated. The identified mediators may also in part act as confounders. Subjects that are more obese or have received dietary advice to change their diet in relation to prescription of anti-hypertensive or lipid-lowering treatment may change their dietary intakes and their level of PA accordingly. However, potentially more important is that, due to social desirability, these subjects may be more likely to misreport such information. This notion is strengthened by the finding that most observed associations were strengthened after exclusion of potential energy mis-reporters.

In conclusion, in this study a diet with low intake of vegetables and fruit, fish and shellfish and high levels of SSB and red and processed meat were associated with higher risk of aIS. Further, subjects with low total PA level, driven by lack of leisure-time PA, were at higher risk of aIS. A high diet quality seemed to have a risk reducing effect regardless of level of PA. In terms of health benefits, these findings suggest that persons with a sedentary lifestyle may still gain some positive health benefits by eating a healthy diet.

## Data availability statement

The data analyzed in this study is subject to the following licenses/restrictions: Datasets analyzed during the current study are not publicly available due to the nature of the sensitive personal data and study materials. However, procedures for sharing data, analytic methods, and study materials for reproducing the results following Swedish legislation can be arranged by contacting the corresponding author or study organization. Requests to access these datasets should be directed to https://www.malmo-kohorter.lu.se/malmo-kost-cancer-mkc.

## Ethics statement

The studies involving human participants were reviewed and approved by Regional Ethical Review Authority Lund, Sweden Dnr § LU 51-90, 2007/166. The patients/participants provided their written informed consent to participate in this study.

## Author contributions

AJ and ID conceived of the concept and design of the work, performed the analyses, interpretation of data, and agreed to be accountable for all aspects of the work. SA and GE contributed with data. AJ drafted the work. SA, ID, PM, ES, and GE revised it critically and approved the final version to be published. All authors contributed to the article and approved the submitted version.
