# Supplementary material for: Components of a healthy diet and different types of physical activity and risk of atherothrombotic ischemic stroke: A prospective cohort study
Source: Front Cardiovasc Med. 2022 Oct 14;9:993112. doi: 10.3389/fcvm.2022.993112 (PMC9614044; doi:10.3389/fcvm.2022.993112)
Supplement: Supplementary file 1 [file Data_Sheet_1.docx]

***Supplementary material***

MDCS Participants (N = 30,446)

with baseline examinations 1991-1996

Prevalent ischemic stroke excluded (N = 233)

Prevalent atrial fibrillation/flutter excluded (N = 312)

Prevalent atherosclerotic disease excluded (N = 785)

Included participants (N = 23,797) with complete follow-up until 31 December 2016

cdcc

Prevalent diabetes mellitus excluded (N = 1,380)

Incident atherothrombotic ischemic stroke (N = 1,937)

No incident atherothrombotic ischemic stroke (N = 21,860)

xx

Subjects with implausible values or missing data on included covariates excluded (N = 4,739)

Figure S1. Descriptive flow diagram of study population with exclusions. Some participants fulfilled multiple exclusion criteria.


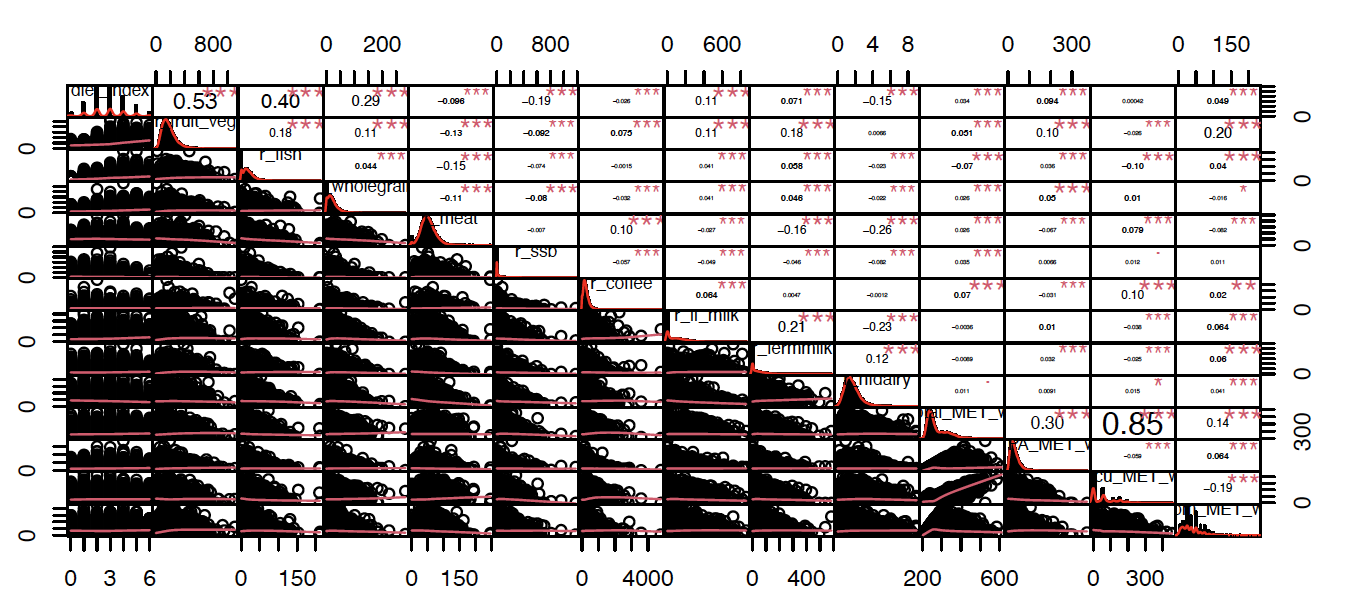


Figure S2. Description of energy adjusted dietary variables and physical activity measures examined in the present study. The figure shows the histogram distribution of each variable on the diagonal. The bivariate scatter plots with a fitted line are shown on the bottom of the diagonal. The bivariate correlation coefficients with corresponding significance level (***<0.0001, **<0.001, *<0.01) are shown on the top of the diagonal.


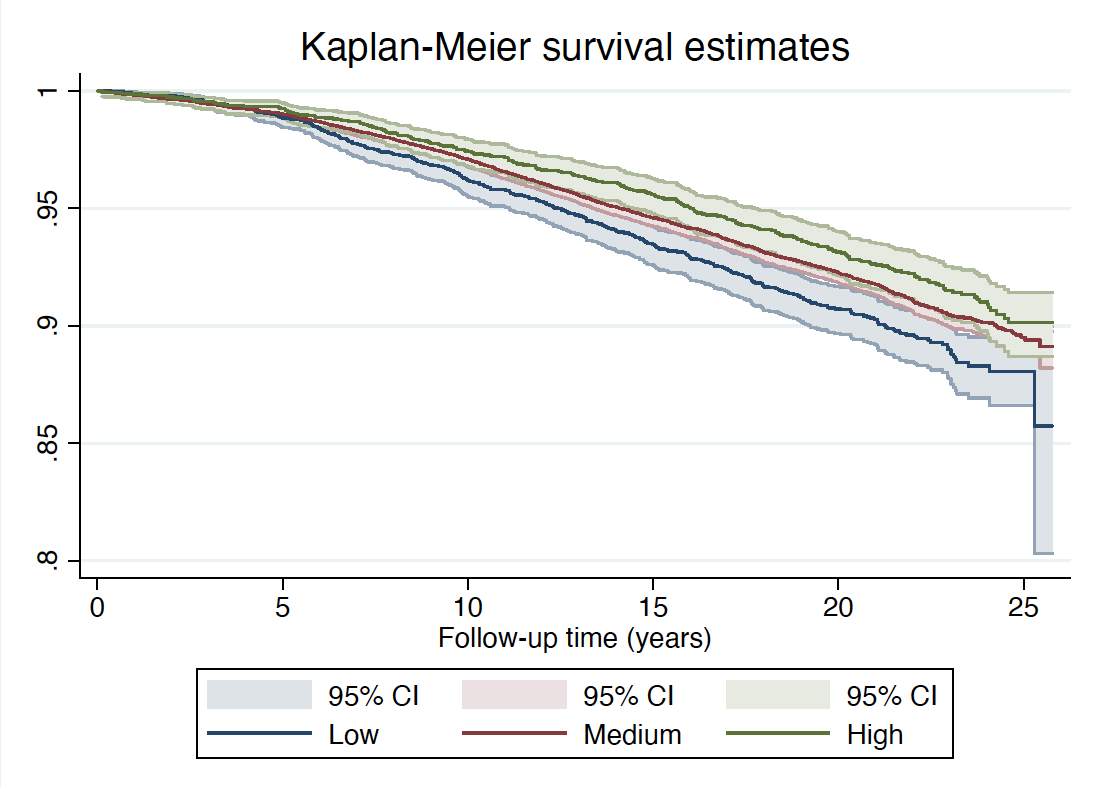


# Figure S3. Kaplan-Meier survival curves showing the estimated disease-free survival probability for atherothrombotic ischemic stroke by categories of overall diet quality.


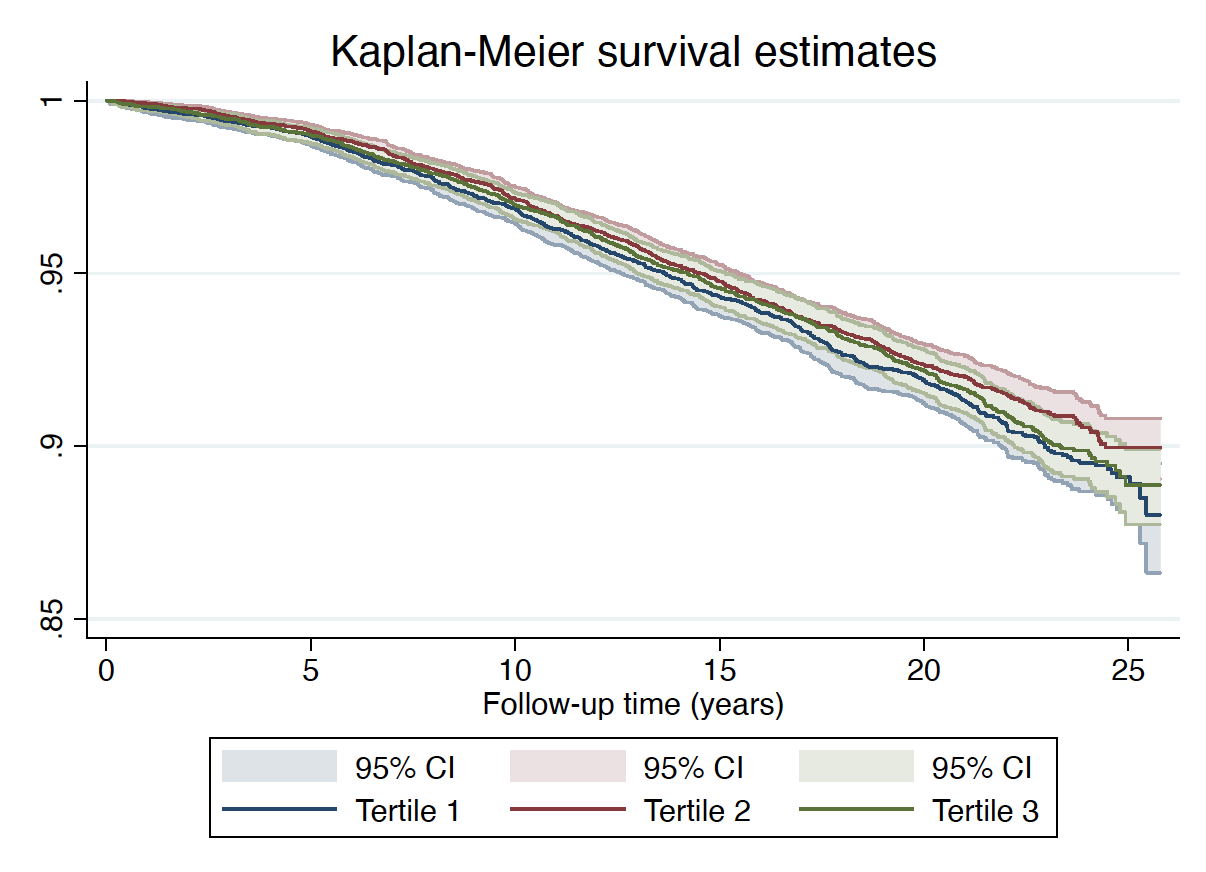


# Figure S4. Kaplan-Meier survival curves showing the estimated disease-free survival probability for atherothrombotic ischemic stroke by tertiles of leisure-time physical activity.

Table S1. Hazard ratios (HR) and 95% confidence intervals (CI) for atherothrombotic ischemic stroke by overall diet quality and dietary components in the Malmö Diet and Cancer Study (n=23,797).

|  | Diet quality index | | | |  |  |  |
| --- | --- | --- | --- | --- | --- | --- | --- |
|  | Low | Medium | High | *p* trend |  | Per point increase | *p* value |
| Subjects/cases | 3702/341 | 17004/1363 | 3091/1937 | - |  | - | - |
| Age and sex adjusted model | Ref | 0.82 (0.73-0.92) | 0.73 (0.62-0.87) | 1.6 x 10^-4^ |  | 0.95 (0.91-0.98) | 7.3 x 10^-4^ |
| Multivariable model 1 | Ref | 0.87 (0.77-0.97) | 0.82 (0.69-0.97) | 0.015 |  | 0.97 (0.94-1.00) | 0.087 |
| Sensitivity analysis | Ref | 0.83 (0.73-0.95) | 0.76 (0.63-0.91) | 0.0019 |  | 0.96 (0.93-1.00) | 0.029 |
| Multivariable model 2 | Ref | 0.86 (0.77-0.97) | 0.80 (0.68-0.95) | 0.0080 |  | 0.97 (0.94-1.00) | 0.045 |
| Sensitivity analysis | Ref | 0.83 (0.73-0.95) | 0.74 (0.62-0.90) | 0.0011 |  | 0.96 (0.92-0.99) | 0.017 |
|  | Fruit and vegetables (g/day per 1000 kcal) | | | |  |  |  |
|  | Q1 | Q2 | Q3 | Q4 | *p* trend | Per SD increase | *p* value |
| Subjects/cases | 5953/561 | 5954/504 | 5950/446 | 5940/426 | - |  |  |
| Age and sex adjusted model | Ref | 0.89 (0.79-1.00) | 0.77 (0.67-0.87) | 0.80 (0.70-0.92) | 0.0014 | 0.92 (0.87-0.97) | 1.6 x 10^-3^ |
| Multivariable model 1 | Ref | 0.95 (0.84-1.07) | 0.85 (0.74-0.96) | 0.91 (0.79-1.04) | 0.056 | 0.96 (0.91-1.01) | 0.15 |
| Sensitivity analysis | Ref | 0.95 (0.83-1.08) | 0.86 (0.75-0.99) | 0.87 (0.74-1.02) | 0.031 | 0.93 (0.87-0.99) | 0.019 |
| Multivariable model 2 | Ref | 0.94 (0.83-1.06) | 0.84 (0.73-0.95) | 0.88 (0.77-1.01) | 0.026 | 0.96 (0.91-1.01) | 0.087 |
| Sensitivity analysis | Ref | 0.95 (0.83-1.08) | 0.86 (0.74-0.99) | 0.86 (0.73-1.00) | 0.020 | 0.92 (0.87-0.98) | 0.014 |
|  | Fish and shellfish (g/day per 1000 kcal) | | | |  |  |  |
|  | Q1 | Q2 | Q3 | Q4 | *p* trend | Per SD increase | *p* value |
| Subjects/cases | 5949/482 | 5944/476 | 5961/483 | 5943/496 | - |  |  |
| Age and sex adjusted model | Ref | 0.86 (0.76-0.98) | 0.84 (0.74-0.95) | 0.85 (0.74-0.96) | 0.012 | 0.94 (0.90-0.99) | 0.018 |
| Multivariable model 1 | Ref | 0.88 (0.77-0.99) | 0.86 (0.76-0.98) | 0.88 (0.78-1.00) | 0.073 | 0.96 (0.91-1.01) | 0.080 |
| Sensitivity analysis | Ref | 0.91 (0.79-1.04) | 0.88 (0.77-1.02) | 0.86 (0.75-1.00) | 0.044 | 0.95 (0.90-1.00) | 0.046 |
| Multivariable model 2 | Ref | 0.86 (0.76-0.98) | 0.86 (0.76-0.98) | 0.87 (0.77-0.99) | 0.051 | 0.95 (0.91-1.00) | 0.046 |
| Sensitivity analysis | Ref | 0.90 (0.78-1.03) | 0.88 (0.77-1.02) | 0.86 (0.74-0.99) | 0.041 | 0.94 (0.89-1.00) | 0.034 |
|  | High-fiber bread and cereals (g/day per 1000 kcal) | | | |  |  |  |
|  | Q1 | Q2 | Q3 | Q4 | *p* trend | Per SD increase | *p* value |
| Subjects/cases | 5950/511 | 5949/448 | 5949/462 | 5949/516 | - |  |  |
| Age and sex adjusted model | Ref | 0.82 (0.73-0.94) | 0.82 (0.72-0.93) | 0.86 (0.76-0.97) | 0.023 | 0.96 (0.91-1.00) | 0.069 |
| Multivariable model 1 | Ref | 0.87 (0.76-0.98) | 0.87 (0.76-0.98) | 0.93 (0.82-1.05) | 0.27 | 0.98 (0.94-1.03) | 0.41 |
| Sensitivity analysis | Ref | 0.91 (0.80-1.05) | 0.85 (0.74-0.98) | 0.92 (0.80-1.05) | 0.16 | 0.97 (0.92-1.02) | 0.30 |
| Multivariable model 2 | Ref | 0.86 (0.76-0.98) | 0.86 (0.76-0.98) | 0.93 (0.82-1.05) | 0.27 | 0.98 (0.94-1.03) | 0.44 |
| Sensitivity analysis | Ref | 0.91 (0.79-1.05) | 0.85 (0.74-0.98) | 0.92 (0.81-1.06) | 0.18 | 0.98 (0.93-1.03) | 0.34 |

|  | Red and processed meat (g/day per 1000 kcal) | | | |  |  |  |
| --- | --- | --- | --- | --- | --- | --- | --- |
|  | Q1 | Q2 | Q3 | Q4 | *p* trend | Per SD increase | *p* value |
| Subjects/cases | 5944/445 | 5953/488 | 5951/493 | 5949/511 | - |  |  |
| Age and sex adjusted model | Ref | 1.08 (0.95-1.22) | 1.12 (0.99-1.28) | 1.25 (1.10-1.42) | 6.6 x 10^-4^ | 1.08 (1.04-1.13) | 4.9 x 10^-4^ |
| Multivariable model 1 | Ref | 1.04 (0.92-1.19) | 1.07 (0.94-1.22) | 1.16 (1.01-1.32) | 0.028 | 1.05 (1.01-1.10) | 0.026 |
| Sensitivity analysis | Ref | 1.07 (0.93-1.23) | 1.13 (0.98-1.30) | 1.23 (1.07-1.43) | 0.0031 | 1.08 (1.03-1.14) | 0.0026 |
| Multivariable model 2 | Ref | 1.02 (0.90-1.17) | 1.04 (0.92-1.19) | 1.12 (0.98-1.28) | 0.097 | 1.04 (0.99-1.09) | 0.10 |
| Sensitivity analysis | Ref | 1.05 (0.91-1.21) | 1.10 (0.95-1.27) | 1.19 (1.03-1.38) | 0.014 | 1.07 (1.01-1.13) | 0.013 |
|  | Sugar-sweetened beverages (g/day per 1000 kcal) | | | |  |  |  |
|  | Q1 | Q2 | Q3 | Q4 | *p* trend | Per SD increase | *p* value |
| Subjects/cases | 10741/894 | 4352/303 | 4352/361 | 4352/379 | - |  |  |
| Age and sex adjusted model | Ref | 0.80 (0.70-0.91) | 1.03 (0.91-1.17) | 1.07 (0.95-1.21) | 0.21 | 1.07 (1.03-1.11) | 8.0 x 10^-4^ |
| Multivariable model 1 | Ref | 0.81 (0.71-0.93) | 1.03 (0.91-1.17) | 1.06 (0.94-1.20) | 0.27 | 1.06 (1.02-1.11) | 2.5 x 10^-3^ |
| Sensitivity analysis | Ref | 0.81 (0.71-0.94) | 1.01 (0.88-1.15) | 1.05 (0.92-1.20) | 0.41 | 1.06 (1.01-1.11) | 9.1 x 10^-3^ |
| Multivariable model 2 | Ref | 0.81 (0.71-0.93) | 1.03 (0.91-1.16) | 1.04 (0.92-1.18) | 0.38 | 1.06 (1.02-1.10) | 4.7 x 10^-3^ |
| Sensitivity analysis | Ref | 0.81 (0.70-0.93) | 1.00 (0.88-1.15) | 1.04 (0.91-1.18) | 0.53 | 1.06 (1.01-1.11) | 0.013 |
|  | Coffee (g/day per 1000 kcal) | | | |  |  |  |
|  | Q1 | Q2 | Q3 | Q4 | *p* trend | Per SD increase | *p* value |
| Subjects/cases | 5950/488 | 5949/500 | 5949/507 | 5949/442 |  |  |  |
| Age and sex adjusted model | Ref | 1.02 (0.90-1.15) | 1.14 (1.01-1.29) | 1.23 (1.08-1.40) | 6.4 x 10^-4^ | 1.09 (1.05-1.13) | 1.3 x 10^-5^ |
| Multivariable model 1 | Ref | 0.98 (0.86-1.11) | 1.08 (0.95-1.22) | 1.09 (0.96-1.25) | 0.090 | 1.04 (0.99-1.09) | 0.15 |
| Sensitivity analysis | Ref | 0.98 (0.86-1.12) | 1.11 (0.97-1.27) | 1.11 (0.95-1.28) | 0.072 | 1.04 (0.97-1.10) | 0.27 |
| Multivariable model 2 | Ref | 0.97 (0.86-1.10) | 1.07 (0.94-1.21) | 1.09 (0.95-1.24) | 0.11 | 1.03 (0.98-1.08) | 0.20 |
| Sensitivity analysis | Ref | 0.98 (0.85-1.12) | 1.10 (0.96-1.26) | 1.11 (0.95-1.29) | 0.074 | 1.03 (0.97-1.10) | 0.30 |
|  | Low-fat milk products (g/day per 1000 kcal) | | | |  |  |  |
|  | Q1 | Q2 | Q3 | Q4 | *p* trend | Per SD increase | *p* value |
| Subjects/cases | 5950/511 | 5949/489 | 5049/435 | 5949/502 |  |  |  |
| Age and sex adjusted model | Ref | 1.01 (0.89-1.14) | 0.84 (0.74-0.96) | 0.99 (0.87-1.12) | 0.30 | 1.01 (0.96-1.05) | 0.79 |
| Multivariable model 1 | Ref | 1.05 (0.93-1.19) | 0.89 (0.78-1.01) | 1.02 (0.90-1.16) | 0.67 | 1.01 (0.97-1.06) | 0.59 |
| Sensitivity analysis | Ref | 1.00 (0.87-1.14) | 0.84 (0.73-0.96) | 0.99 (0.87-1.14) | 0.39 | 1.00 (0.95-1.05) | 0.95 |
| Multivariable model 2 | Ref | 1.05 (0.93-1.19) | 0.88 (0.77-1.00) | 1.00 (0.88-1.13) | 0.39 | 1.00 (0.96-1.05) | 0.98 |
| Sensitivity analysis | Ref | 0.99 (0.86-1.13) | 0.83 (0.72-0.95) | 0.97 (0.84-1.11) | 0.21 | 0.99 (0.94-1.04) | 0.63 |
|  | Fermented milk products (g/day per 1000 kcal) | | | |  |  |  |
|  | Q1 | Q2 | Q3 | Q4 | *p* trend | Per SD increase | *p* value |
| Subjects/cases | 8098/742 | 5233/399 | 5233/406 | 5233/390 | - |  |  |
| Age and sex adjusted model | Ref | 0.90 (0.79-1.01) | 0.88 (0.78-1.00) | 0.83 (0.72-0.94) | 0.0022 | 0.94 (0.90-0.99) | 0.015 |
| Multivariable model 1 | Ref | 0.93 (0.83-1.05) | 0.93 (0.82-1.05) | 0.89 (0.79-1.01) | 0.071 | 0.97 (0.92-1.02) | 0.19 |
| Sensitivity analysis | Ref | 0.96 (0.84-1.10) | 0.94 (0.82-1.07) | 0.90 (0.78-1.03) | 0.13 | 0.96 (0.91-1.02) | 0.17 |
| Multivariable model 2 | Ref | 0.94 (0.83-1.06) | 0.93 (0.82-1.05) | 0.91 (0.80-1.03) | 0.12 | 0.97 (0.93-1.02) | 0.29 |
| Sensitivity analysis | Ref | 0.96 (0.84-1.10) | 0.94 (0.82-1.08) | 0.92 (0.80-1.06) | 0.20 | 0.97 (0.92-1.02) | 0.26 |
|  | High-fat dairy products (portions/day per 1000 kcal) | | | |  |  |  |
|  | Q1 | Q2 | Q3 | Q4 | *p* trend | Per SD increase | *p* value |
| Subjects/cases | 5951/532 | 5950/516 | 5951/451 | 5945/438 | - |  |  |
| Age and sex adjusted model | Ref | 1.00 (0.89-1.13) | 0.94 (0.83-1.07) | 0.96 (0.84-1.09) | 0.37 | 0.97 (0.93-1.02) | 0.22 |
| Multivariable model 1 | Ref | 1.02 (0.90-1.15) | 0.98 (0.86-1.11) | 0.99 (0.87-1.12) | 0.68 | 0.98 (0.93-1.02) | 0.36 |
| Sensitivity analysis | Ref | 1.01 (0.89-1.16) | 0.95 (0.83-1.09) | 0.98 (0.85-1.13) | 0.56 | 0.97 (0.92-1.02) | 0.24 |
| Multivariable model 2 | Ref | 1.03 (0.92-1.17) | 1.01 (0.89-1.14) | 1.02 (0.90-1.16) | 0.84 | 0.99 (0.95-1.04) | 0.74 |
| Sensitivity analysis | Ref | 1.03 (0.90-1.18) | 0.98 (0.85-1.12) | 1.01 (0.88-1.17) | 0.93 | 0.98 (0.93-1.03) | 0.47 |

Multivariable model 1: adjusted for age, sex, smoking, and educational level.

Multivariable model 2: model 1 + stroke heredity score, dyslipidemia, hypertension, BMI and total physical activity level.

Sensitivity analysis: energy mis-reporters excluded from study population

Table S2. Hazard ratios (HR) and 95% confidence intervals (CI) for atherothrombotic ischemic stroke by quartiles of total physical activity level (PAL), leisure-time PAL, occupational PAL, and domestic PAL in the Malmö Diet and Cancer Study (n=23,797).

|  | Total PAL (MET-h/week) | | | |  |  |  |
| --- | --- | --- | --- | --- | --- | --- | --- |
|  | Q1 | Q2 | Q3 | Q4 | *p* trend | Per SD increase | *p* value |
| Subjects/cases | 5933/581 | 5952/441 | 5949/525 | 5963/390 | - |  |  |
| Age- and sex-adjusted model | Ref | 0.79 (0.70-0.90) | 0.98 (0.87-1.11) | 0.97 (0.85-1.11) | 0.73 | 1.03 (0.98-1.08) | 0.27 |
| Multivariable model 1 | Ref | 0.82 (0.72-0.93) | 1.00 (0.89-1.13) | 0.95 (0.83-1.09) | 0.91 | 1.01 (0.96-1.07) | 0.60 |
| Sensitivity analysis | Ref | 0.83 (0.73-0.95) | 1.03 (0.90-1.17) | 0.91 (0.77-1.07) | 0.82 | 1.00 (0.94-1.06) | 0.99 |
| Multivariable model 2 | Ref | 0.83 (0.73-0.94) | 1.03 (0.91-1.16) | 0.98 (0.86-1.12) | 0.55 | 1.02 (0.97-1.08) | 0.38 |
| Sensitivity analysis | Ref | 0.84 (0.74-0.96) | 1.06 (0.93-1.21) | 0.95 (0.81-1.12) | 0.67 | 1.02 (0.96-1.09) | 0.54 |
|  | Leisure-time PAL (MET-h/week) | | | |  |  |  |
|  | Q1 | Q2 | Q3 | Q4 | *p* trend | Per SD increase | *p* value |
| Subjects/cases | 5953/533 | 5950/445 | 5956/464 | 5938/495 | - |  |  |
| Age- and sex-adjusted model | Ref | 0.80 (0.71-0.91) | 0.81 (0.72-0.92) | 0.82 (0.72-0.92) | 0.0025 | 0.94 (0.90-0.99) | 0.0096 |
| Multivariable model 1 | Ref | 0.82 (0.72-0.93) | 0.84 (0.75-0.96) | 0.85 (0.75-0.96) | 0.018 | 0.95 (0.91-0.99) | 0.028 |
| Sensitivity analysis | Ref | 0.85 (0.74-0.98) | 0.83 (0.72-0.95) | 0.86 (0.75-0.99) | 0.031 | 0.95 (0.91-1.00) | 0.063 |
| Multivariable model 2 | Ref | 0.84 (0.74-0.95) | 0.86 (0.76-0.98) | 0.87 (0.77-0.99) | 0.062 | 0.96 (0.92-1.01) | 0.089 |
| Sensitivity analysis | Ref | 0.87 (0.76-1.00) | 0.85 (0.74-0.98) | 0.89 (0.78-1.02) | 0.10 | 0.97 (0.92-1.02) | 0.18 |
|  | Domestic PAL (MET-h/week) | | | |  |  |  |
|  | Q1 | Q2 | Q3 | Q4 | *p* trend | Per SD increase | *p* value |
| Subjects/cases | 6040/540 | 6260/488 | 6566/479 | 4931/430 | - |  |  |
| Age- and sex-adjusted model | Ref | 1.01 (0.89-1.15) | 1.00 (0.87-1.15) | 1.08 (0.92-1.25) | 0.40 | 1.02 (0.97-1.07) | 0.44 |
| Multivariable model 1 | Ref | 1.02 (0.90-1.15) | 1.01 (0.87-1.16) | 1.07 (0.92-1.25) | 0.42 | 1.02 (0.97-1.07) | 0.47 |
| Sensitivity analysis | Ref | 0.95 (0.82-1.09) | 0.99 (0.85-1.16) | 1.06 (0.89-1.25) | 0.47 | 1.01 (0.95-1.07) | 0.75 |
| Multivariable model 2 | Ref | 1.02 (0.90-1.16) | 1.01 (0.88-1.16) | 1.06 (0.91-1.24) | 0.49 | 1.01 (0.96-1.07) | 0.59 |
| Sensitivity analysis | Ref | 0.95 (0.83-1.09) | 1.00 (0.86-1.16) | 1.05 (0.89-1.24) | 0.51 | 1.01 (0.95-1.06) | 0.83 |
|  | Occupational PAL (MET-h/week) | | | |  |  |  |
|  | Q1 | Q2 | Q3 | Q4 | *p* trend | Per SD increase | *p* value |
| Subjects/cases | 9370/1031 | 6134/359 | 2391/156 | 5902/391 | - |  |  |
| Age- and sex-adjusted model | Ref | 0.85 (0.74-0.98) | 0.89 (0.74-1.07) | 1.02 (0.88-1.17) | 0.64 | 1.03 (0.97-1.09) | 0.30 |
| Multivariable model 1 | Ref | 0.92 (0.80-1.06) | 0.96 (0.80-1.15) | 1.02 (0.89-1.18) | 0.64 | 1.02 (0.97-1.08) | 0.47 |
| Sensitivity analysis | Ref | 0.92 (0.79-1.07) | 0.94 (0.77-1.15) | 1.00 (0.85-1.18) | 0.97 | 1.01 (0.94-1.08) | 0.88 |
| Multivariable model 2 | Ref | 0.94 (0.81-1.08) | 0.98 (0.81-1.17) | 1.05 (0.91-1.22) | 0.39 | 1.03 (0.97-1.09) | 0.31 |
| Sensitivity analysis | Ref | 0.93 (0.80-1.09) | 0.97 (0.79-1.18) | 1.05 (0.89-1.24) | 0.57 | 1.02 (0.96-1.10) | 0.51 |

Multivariable model 1: adjusted for age, sex, smoking, and educational level.

Multivariable model 2: adjusted for model 1 + stroke heredity, dyslipidemia, hypertension, BMI and diet quality index score.

Sensitivity analysis: energy mis-reporters excluded from study sample.

Table S3. Standardized 20-year incidence rates (%) and 95% confidence intervals of aIS across diet quality and physical activity level categories. Risk estimates based on a Cox regression model and standardized to the mean level of age, sex, educational level, smoking status, stroke heredity, BMI, hypertension, and dyslipidemia after exclusion of energy mis-reporters.

|  | Diet quality | | |
| --- | --- | --- | --- |
| LTPA | Low | Medium | High |
| Low | 8.1 (6.6-9.5) | 7.2 (6.5-7.9) | 6.0 (7.6-4.2) |
| Medium | 8.0 (6.3-9.6) | 6.6 (5.9-7.3) | 5.6 (4.2-7.0) |
| High | 8.1 (6.5-9.8) | 6.2 (5.5-6.8) | 6.1 (4.7-7.4) |
